# The anticancer effects of PCAIs in pancreatic cancer cells involve MAPK and PI3K/AKT pathways hyperactivation

**DOI:** 10.18632/oncotarget.28879

**Published:** 2026-06-03

**Authors:** Kweku Ofosu-Asante, Jassy Mary S. Lazarte, Amarender Goud Burra, Nazarius S. Lamango

**Affiliations:** ^1^Florida A&M University College of Pharmacy Pharmaceutical Sciences, Institute of Public Health, Tallahassee, FL 32307, USA

**Keywords:** PCAIs, PDAC, MAPK, PI3K/AKT, KRAS

## Abstract

There remains an unmet need for effective drugs targeting KRAS-driven cancers. Polyisoprenylated cysteinyl amide inhibitors (PCAIs) were designed to disrupt hyperactive mutant KRAS in cancer. Here, we determined the effects of PCAIs on the viability and downstream mediators of KRAS on pancreatic cancer-derived PANC-1 and MIAPaCa-2 cells. NSL-YHJ-2-45 and NSL-YHJ-2-27 were the most potent of the analogs with EC50 values of 3.6 and 3.8 μM, respectively. NSL-YHJ-2-27 treatment of PANC-1 cells stimulated BRAF, MEK 1/2, ERK 1/2 and p90RSK phosphorylation levels by 64 to 150% while CRAF phosphorylation significantly decreased by 27%. Furthermore, 5 μM NSL-YHJ-2-27 depleted 20 to 61% of the monomeric G-proteins, CDC42, RHOA and RAC 1/2/3 while increasing pAKT (Ser 473) and pAKT (Thr 308) phosphorylation by 72 and 190%, respectively. Reactive oxygen species production significantly increased at 3 μM NSL-YHJ-27 in PANC-1 and MIA PaCa-2 by 2- and 9-fold, respectively. Bulk RNA sequencing analysis revealed that treatment of MIA PaCa-2 cells with 3 μM NSL-YHJ-27 resulted in significant differential expression of 88 genes. NSLYHJ-2-27 at 1 μM inhibited over 90% of pancreatic cancer cell migration. The PCAIs induced apoptosis in both PANC-1 and MIA PaCa-2 3D spheroids while doubling caspase 3/7 activity in PANC-1 cells. Taken together, these data obtained using pancreatic cancer cells with KRAS mutations suggest the ability of the PCAIs to prevent metastasis and tumor growth, strongly indicating their potential to serve as effective targeted therapies for treating cancer types driven by the multiple mutant forms of KRAS.

## INTRODUCTION

The RAS family of genes, including KRAS, NRAS, and HRAS, plays a pivotal role in cellular signaling pathways that regulate cell growth, differentiation, and survival [1]. Among them, KRAS is the most mutated in various cancers. These proteins act as molecular switches, cycling between GTP-bound active and GDP-bound inactive states as they play critical signal transduction roles from cell surface receptors to intracellular effectors [2]. KRAS mutations are diverse, with specific variants associated with different types of cancer and clinical outcomes [3]. The most common mutations are on codons 12, 13, and 61 that constitute hotspots for point mutations. Among these, G12C, G12D, G12V, and G13D are the most frequently observed. The mutations impair GTP hydrolysis, leading to constitutive activation of KRAS signaling that result in uncontrolled cell proliferation and tumorigenesis [4, 5].

The mutations drive approximately 30% of all human cancers, making them one of the most significant targets in oncological research [6, 7]. Specifically, KRAS mutations are observed in nearly 25% of all cancers diagnosed in the U.S, with prevalence as high as 90% of pancreatic adenocarcinomas, 40% of colorectal cancers, and 36% of non-small cell lung cancers (NSCLC) [3, 7, 8]. These mutations drive the aggressive nature of the disease, largely due to the limited treatment options and resistance to therapies that target only a subset of mutant forms. This highlights the urgent need for novel pan-KRAS targeted therapies.

A long-standing therapeutic strategy has involved targeting the posttranslational modifications of KRAS, notably the polyisoprenylation that involves the covalent attachment of a *trans*, *trans*farnesyl or an *all trans*-geranylgeranyl isoprenoid group to the C-terminal of KRAS [9, 10]. This modification is essential for chaperone-mediated KRAS localization and anchoring to the inner surface of the plasma membrane to facilitate interactions with upstream receptor tyrosine kinase activators and downstream effectors [11, 12] such as the RAF-MEK-ERK and PI3K-AKT cascades, that regulate cell proliferation and survival [13–17]. Inhibition of polyisoprenylation [10] or delocalization of mutant KRAS with the membrane association inhibitor, *S-trans*, farnesylthiosalicylic acid, (Salirasib) has been shown to disrupt KRAS signaling resulting in tumor cell death [18]. The polyisoprenylated cysteinyl amide inhibitors (PCAIs) are novel agents developed in our lab to mimic the polyisoprenylation of KRAS and mitigate the excessive signaling of mutant KRAS proteins [19, 20].

KRAS signaling through the MAPK and PI3K/AKT pathways implies that hyperactivity of mutant KRAS proteins are the principal drivers of Pancreatic Ductal Adenocarcinoma (PDAC) progression [21]. The two pathways have an interdependence such that inhibition of one result in activation of the other [22, 23]. Mutant KRAS activates the MAP kinase signaling cascade in a manner that results in excessive cell proliferation and survival, tumor invasion and metastasis [24]. Abnormal PI3K/AKT activation promotes carcinogenesis, playing a vital role in the resistance to drugs such as Idelalisib (GS-1101), a selective PI3Kδ inhibitor, in many types of neoplasia including PDAC [25]. The mutant RAS/PI3K interaction is essential for cancer cell survival and proliferation [26]. The inhibition of upstream or downstream effectors to mitigate abnormal MAPK and PI3K/AKT signaling has uncovered new therapeutic targets [27]. Therapies such as the ERK inhibitor, Ulixertinib, and AKT inhibitor, Ipatasertib have been developed to target downstream mediators of mutant KRAS. However, these therapies tend to lose efficacy after prolonged use in PDAC therapy [28–31].

Although mutant KRAS-induced activation of MAPK and P3K/AKT pathways promotes cancer progression, depending on cell type and stimuli, the activation may promote or inhibit apoptosis [32]. Cancer cells must maintain certain levels of MAPK or PI3K/AKT signaling to drive tumor development and progression, but apoptosis ensues when a certain threshold of hyperactivity is exceeded [33, 34]. Hyperactivation of MAPK effectors such as MEK1/2 and ERK1/2 have been shown to stimulate pro-apoptotic effects resulting in cancer cell death [1, 34]. Ecker et al, observed that prolonged overstimulation of AKT increases oxidative stress, rendering chronic lymphocytic leukemia cells susceptible to reactive oxygen species-induced death [1]. Proapoptotic proteins such as the BH3-only BAX, BAD and BIK are also induced in response to prolonged activation of MAPK and PI3K/AKT effectors [35].

Adagrasib and Sotorasib represent a significant breakthrough in the treatment of mutant KRAS^G12C^-driven cancers. For nearly four decades, researchers have sought anti-KRAS therapies since the discovery of the KRAS oncogene, which plays a key role in cancers such as non-small cell lung cancer (NSCLC), colorectal cancer, and pancreatic cancer [36]. Despite their clinical efficacy, the emergence of intrinsic or acquired resistance to these therapies have diminished their long-term treatment benefits. Der et al, observed a reduction in efficacy of Sotorasib in the non-small cell lung cells, NCI-H358 and NCI-H23 after approximately 18 months of prolonged treatment [37].

Although extensive research has established the central role of *KRAS* mutations in pancreatic, colorectal, and lung cancer progression, current therapeutic strategies remain highly limited, particularly due to the diversity of *KRAS* mutant forms and the complex signaling behaviors of the resulting mutant proteins [37, 38]. Besides the emergence of *KRAS* mutations such as *KRAS^G12D^, KRAS^G12V^* and *KRAS^G13R^* against which Adagrasib and Sotorasib are ineffective, research has revealed that treatment with these KRAS^G12C^-targeting drugs results in the hyperactivation of wild type KRAS to compensate for the inhibited KRAS^G12C^ activities [38–40]. Thus, a critical research gap exists in developing broad-spectrum pan-mutant KRAS targeting therapies that effectively suppress multiple mutant KRAS hyperactivities as well as hyperactivated wild-type KRAS, overcoming drug resistance, and disrupting aberrant signaling without triggering compensatory survival pathways [6]. In view of this lack of effective therapies targeting the multiple mutant KRAS oncoprotein forms, this manuscript focuses on the anticancer potential of the PCAIs to suppress the hyperactivated mutant KRAS and wild type KRAS.

## RESULTS

### PCAIs inhibit the viability of pancreatic cancer cells

**Figure 1:**
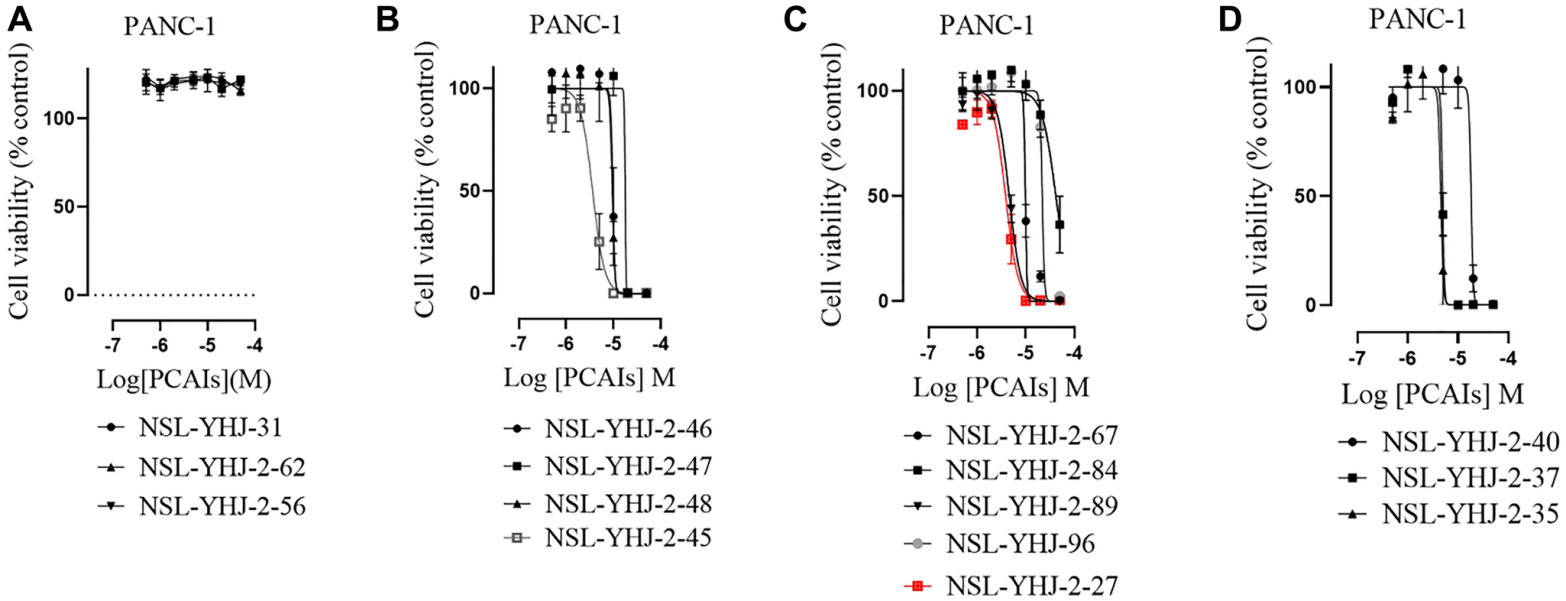
PCAIs inhibit the viability of PANC-1 cancer cells. The effect of the PCAIs on PANC-1 cells was determined after 48 h treatment using the resazurin reduction assay. Concentration-response curves for (**A**) Analogs of PCAIs lacking the S-farnesyl group, (**B**) PCAIs with varying cycloalkyl group sizes, (**C**) PCAIs with different N-terminal ionizable groups/linker sizes compared with the most potent compound (in red), and (**D**) Compounds with varying linker chain lengths.

As earlier stated, posttranslational farnesylation of KRAS is essential for its functional localization in cells [12, 41]. The PCAIs were designed to mimic the modifications and compete against the functional interactions they mediate. Out of the 15 PCAIs analogs tested, NSL-YHJ-2-27 and NSL-YHJ-2-45 were the most potent, with EC_50_ values of 3.8 and 3.6 μM, respectively (Figure 1 and Table 1). An examination of the structures versus activities (structure-activity relationships, SARs) of the PCAIs clearly reveal the importance of the S-farnesyl moiety to the potency against the MIA PaCa-2 and PANC-1 cell viability as NSL-YHJ-2-31, NSL-YHJ-2-62 and NSL-YHJ-2-56 which lack the S-farnesyl tail were ineffective, with EC_50_ values greater than 50 μM. The potency of PCAIs decreased with increasing hydrocarbon link to the 4-methylpiperazinyl ionizable amino groups. This is observed in NSL-YHJ-2-35, NSL-YHJ-2-37 and NSL-YHJ-2-40, with increasing hydrocarbon linker chains resulting in decreasing potency, with EC_50_ values of 4.5, 4.9 and 18 μM, respectively (Table 1). Reduced potency was observed with NSL-YHJ-096 that lacks a spacer between the L-cysteine and an ionizable group at the end of it. Although the α-amino group is ionizable, its spatial localization and strength of the potential electrostatic interactions may be significantly less than for PCAIs with the spacer and the 4-methylpiperazinyl ionizable groups. PCAIs with large N-cycloalkyl substituents such as NSL-YHJ-2-27 with a cyclooctyl moiety were more effective than those with smaller rings as in NSL-YHJ-2-48 with a cyclopropyl group (EC_50_ = 9.4). Due to its superior effectiveness against PANC-1 and MIA PaCa-2 cells [19], NSL-YHJ-2-27 (in red) was chosen for further experiments.

**Table 1:** PCAIs inhibit PANC-1 cell viability

Compound	Structure	EC50 (μM)
NSL-YHJ-096	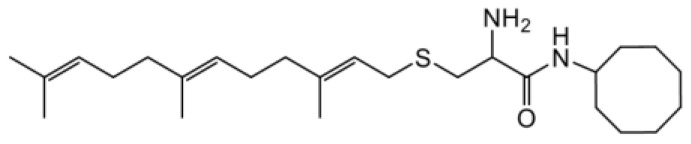	21
NSL-YHJ-2 CONTROL	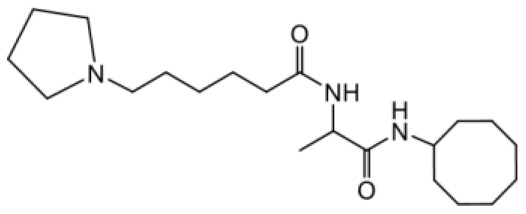	>50
NSL-YHJ-2-62 CONTROL	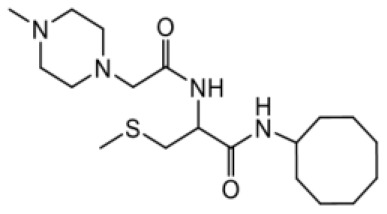	>50
NSL-YHJ-2-56 CONTROL	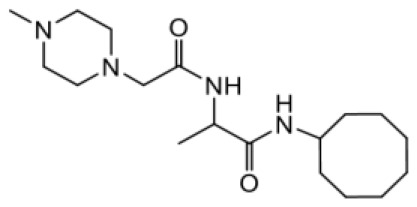	>50
NSL-YHJ-2-40	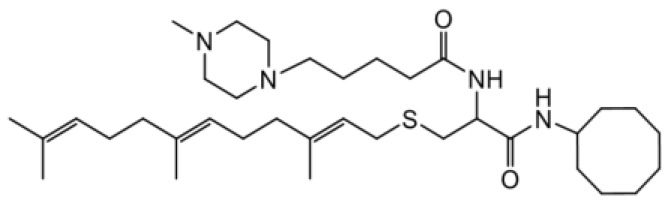	18
NSL-YHJ-2-37	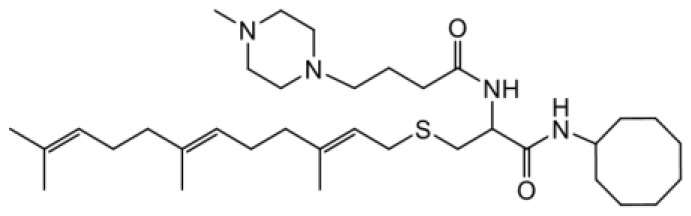	4.9
NSL-YHJ-2-35	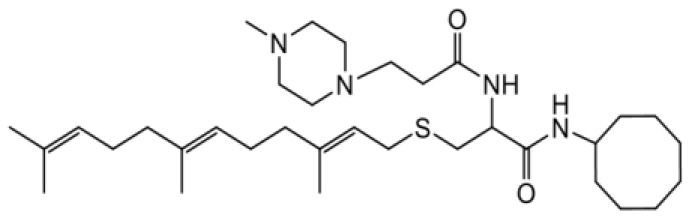	4.5
NSL-YHJ-2-27	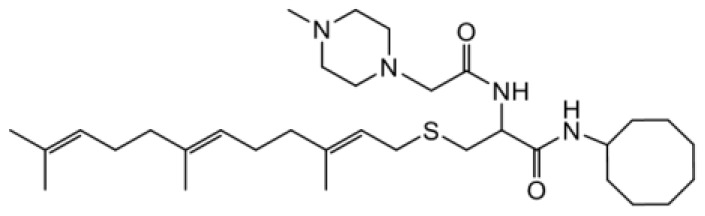	3.8
NSL-YHJ-2-45	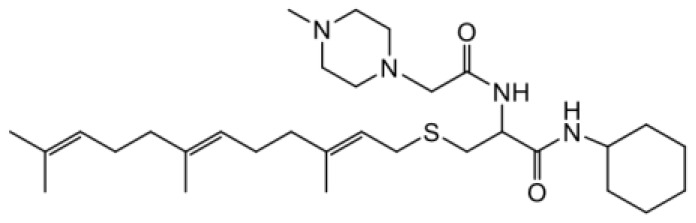	3.6
NSL-YHJ-2-46	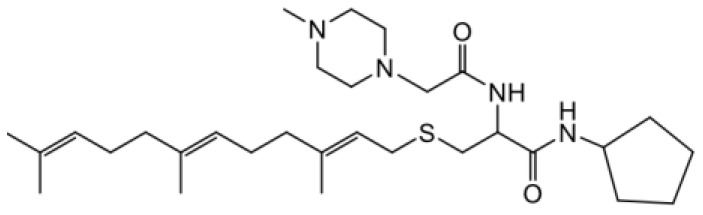	9.7
NSL-YHJ-2-47	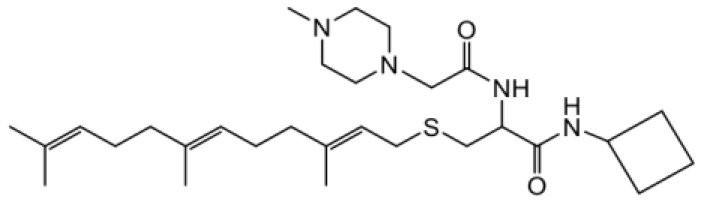	17
NSL-YHJ-2-48	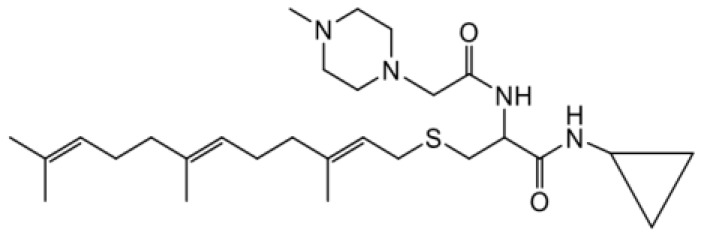	9.1
NSL-YHJ-2-67	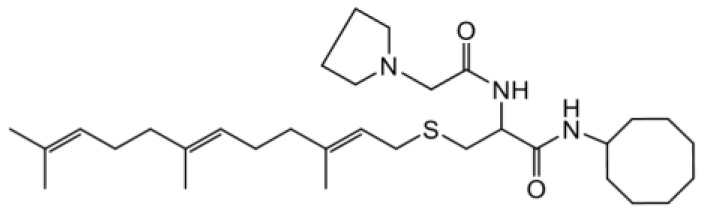	9.8
NSL-YHJ-2-89	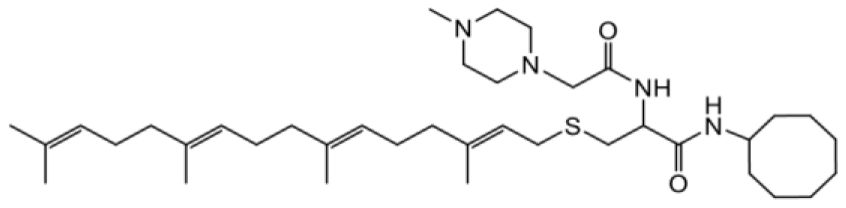	4.5
NSL-YHJ-2-84	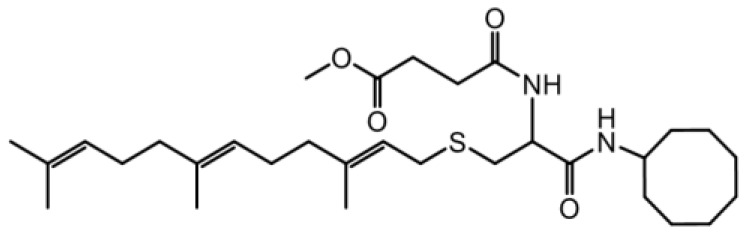	41

PANC-1 cells were treated twice with varying concentrations of 15 different PCAIs analogs over a 48 h period to determine their effectiveness against the viability of PANC-1 cells.

### PCAIs attenuate RHOA and RAC1 monomeric G proteins in PANC-1 cells

**Figure 2:**
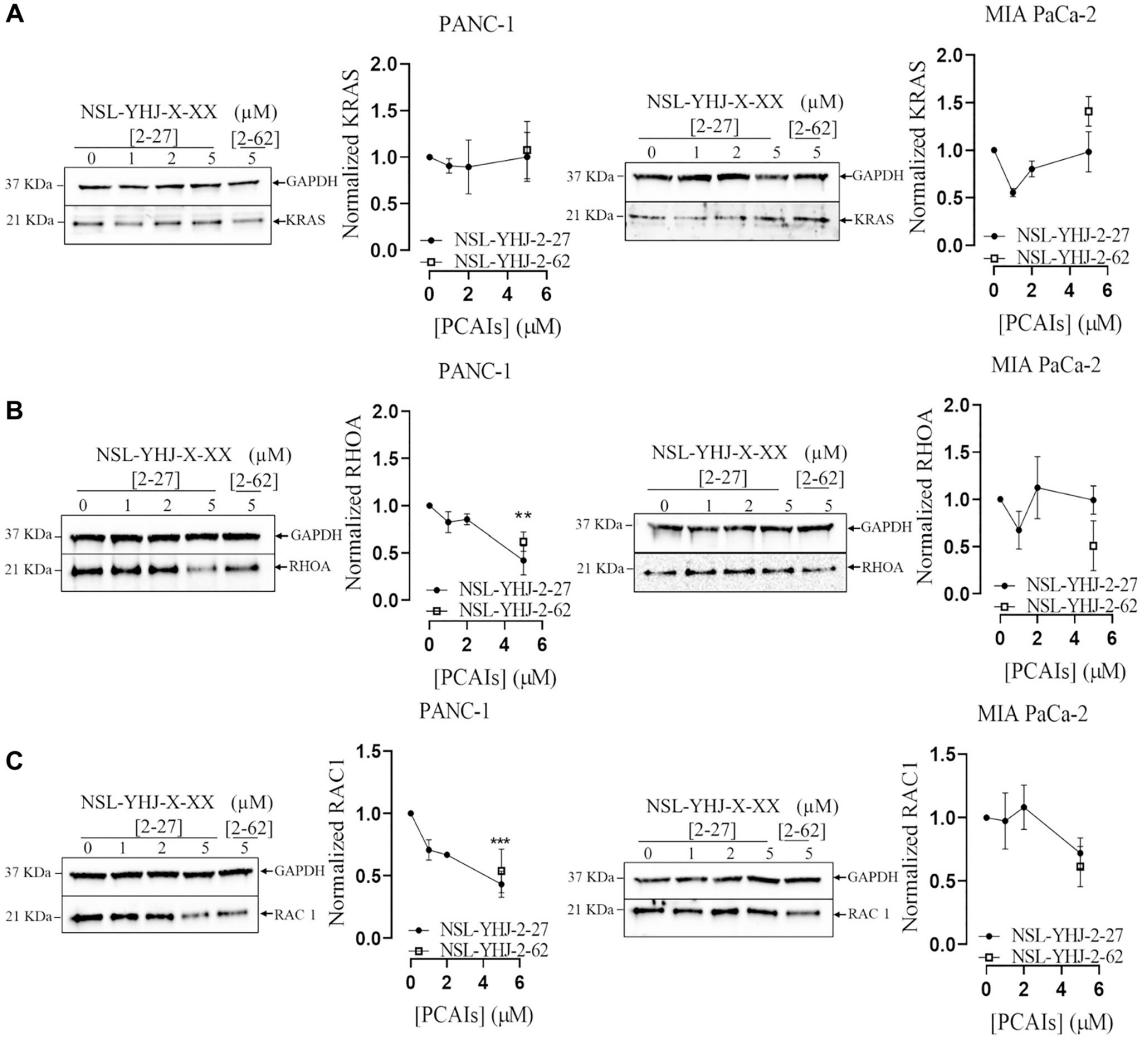
PCAIs decrease the levels of RHOA and RAC1 in PANC-1 cells. (**A**–**C**) Cells were treated for 48 h either with 0–5 μM of NSL-YHJ-2-27 (X-XX is 2-27) or 5 μM of the non-farnesylated analog, NSL-YHJ-2-62 (X-XX is 2-62). They were then lysed and analyzed by western blotting for the effect of PCAIs on protein levels. Western blot band densitometry intensities were determined using Image Lab 6.0 Software and normalized against GAPDH and plotted against the concentrations used. Data are representative of three independent experiments. Statistical significance (^*^p < 0.05, ^**^p < 0.01, and ^***^p < 0.001) was determined by One-Way ANOVA with post hoc Dunnett’s tests.

Monomeric G-proteins play crucial roles in tumorigenesis, angiogenesis and metastasis [42]. Due to these pertinent roles that G-proteins play in cancer, we determined the effect of PCAIs on the levels of these proteins in MIA PaCA-2 and PANC-1 cells. No significant effect was observed on the levels of KRAS in both PANC-1 and MIA PaCa-2 cells after exposure to PCAIs (Figure 2A). At 5 μM NSL-YHJ-2-27, there were significant 71 and 58% decreases in RHOA and RAC1 levels in PANC-1 cells, respectively. However, for MIA PaCa-2, the PCAIs had no significant effect on RHOA levels, but significantly decreased RAC1 levels by 30% (Figure 2B, 2C).

### PCAIs stimulate the phosphorylation activation of MAPK pathway enzymes

**Figure 3:**
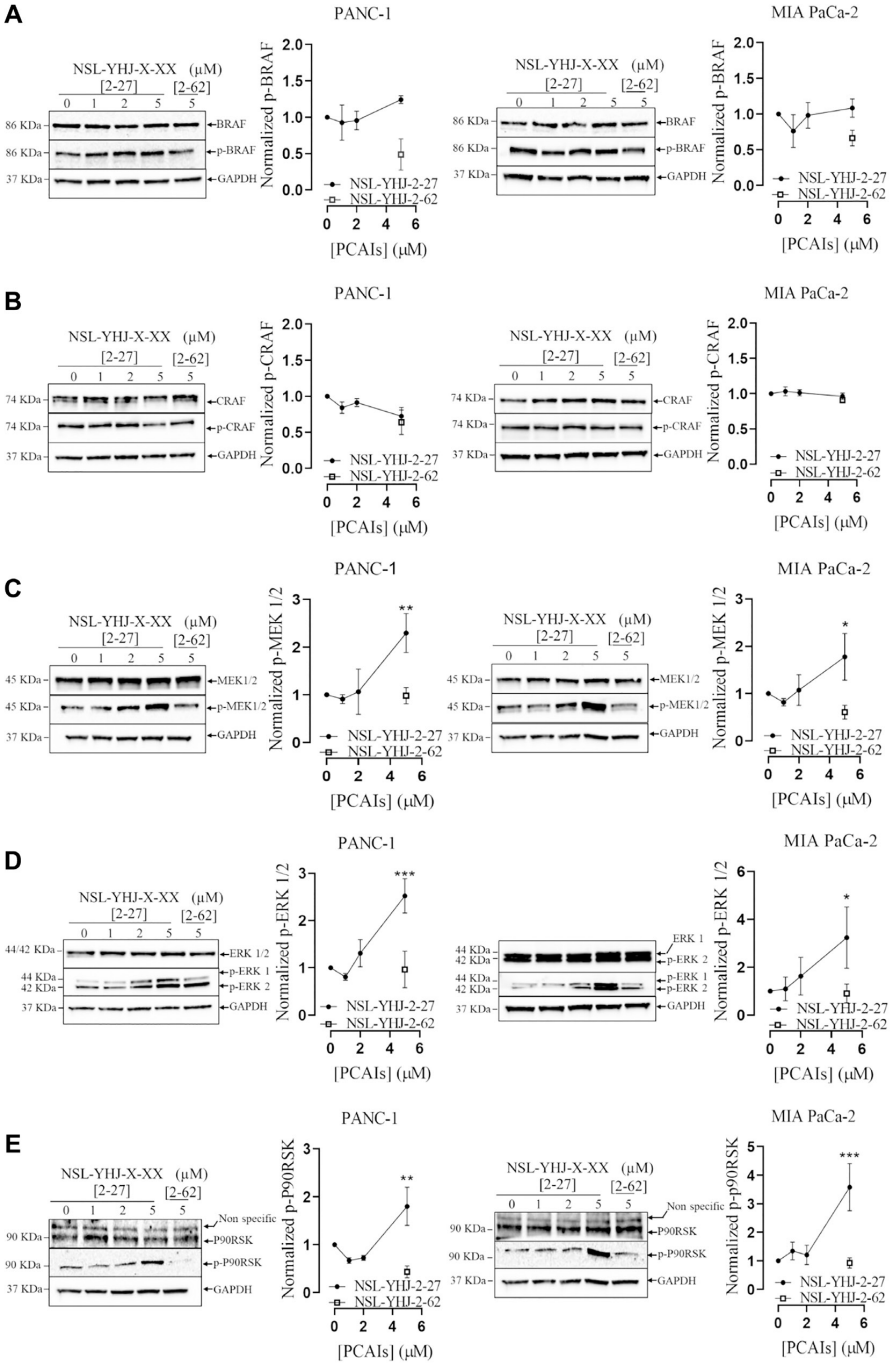
PCAIs stimulate MAPK protein phosphorylation. (**A**–**E**) PANC-1 and MIA PaCa-2 cells were treated with 0–5 μM of NSL-YHJ-2-27 (x-xx is 2-27) and 5 μM of its non-farnesylated control analog, NSL-YHJ-2-62 (x-xx is 2-62) for 48 h. The treated cells were lysed and analyzed by western blotting for MAPK total and phosphorylated protein levels in PANC-1 and MIA PaCa-2 cells. Western blot images and densitometry plots were obtained using Image Lab 6.0 Software normalized against GAPDH and total protein. Statistical significance (^*^p < 0.05, ^**^p < 0.01, and ^***^p < 0.001) was determined with One-Way ANOVA with post hoc Dunnett’s test.

The MAPK pathway is one of the major growth signaling pathways downstream of KRAS that is hyper-stimulated in mutant KRAS-driven pancreatic cancers [43]. To further understand the mechanistic effects of PCAIs on PANC-1 and MIA PaCa-2 cells, we investigated the effects of the PCAIs treatment on the activation of proteins involved in downstream signaling of KRAS by western blotting (Figure 3). No significant changes in the phosphorylation of BRAF (Figure 3A) and CRAF (Figure 3B), were observed in either cell line after exposure to NSL-YHJ-2-27 for 48 h. Significant activation of MEK, ERK and P90RSK phosphorylation was observed in both cell lines. At 5 μM NSL-YHJ-2-27, p-MEK 1/2 phosphorylation levels in PANC-1 and MIA PaCa-2 were elevated by 129 and 78% (Figure 3C), respectively. Also, p-ERK1/2 (Figure 3D) and pP90RSK (Figure 3E) levels in PANC-1 cells increased by 150 and 79%, and in MIA PaCa-2 by 270 and 250%, respectively.

### PCAIs stimulate the phosphorylation of AKT

**Figure 4:**
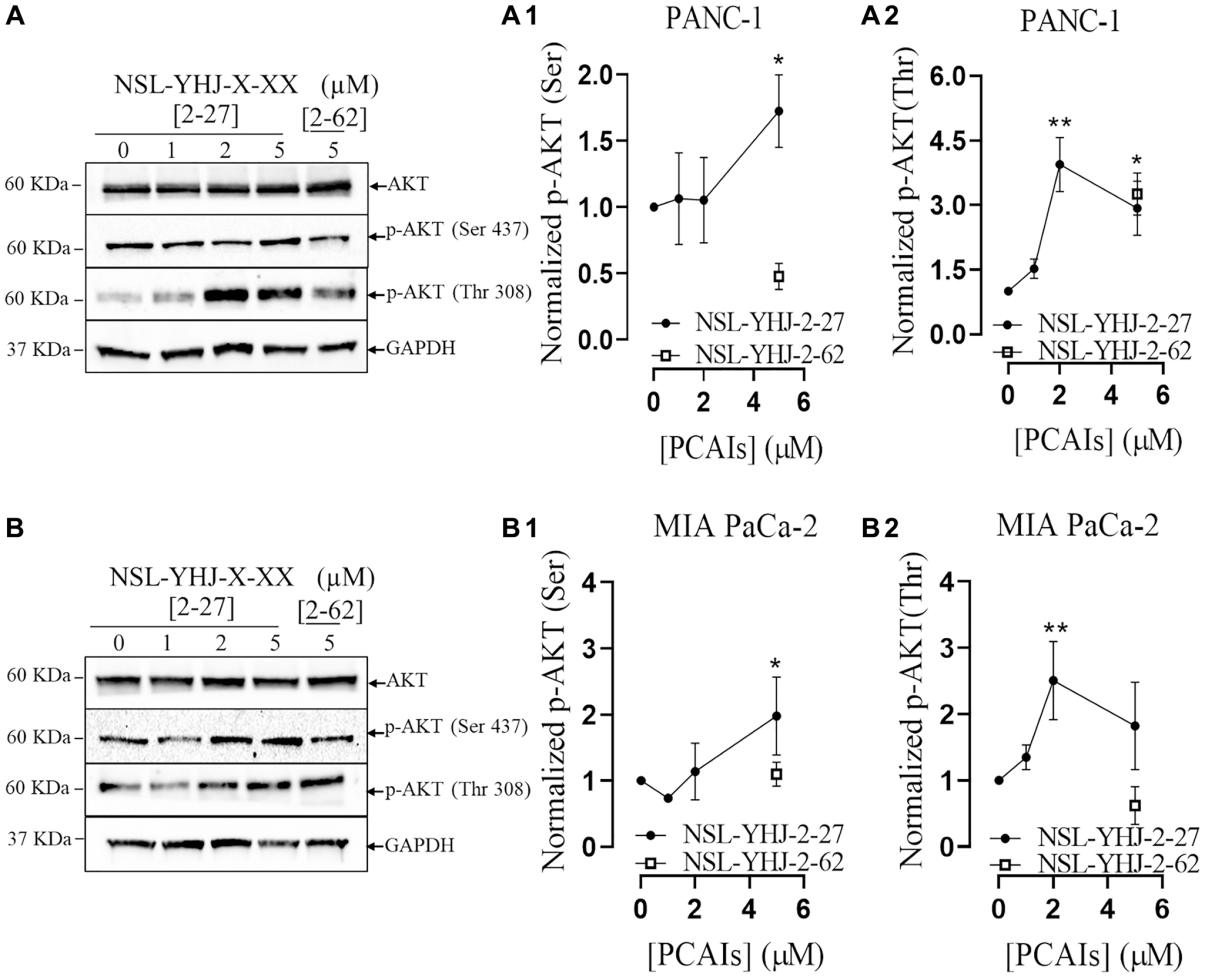
PCAIs stimulate the phosphorylation of the PI3K/AKT pathway proteins. Cells were treated either with 0–5 μM of NSL-YHJ-2-27 (X-XX is 2-27) or 5 μM of the non-farnesylated analog, NSL-YHJ-2-62 (X-XX is 2-62) for 48 h, lysed and analyzed by western blot for the effect of PCAIs on total protein levels as well as for the levels of phosphorylated AKT (Ser473) and AKT (Thr308) in both (**A**, **A1**, **A2**) PANC-1 and (**B**, **B1**, **B2**) MIAPaCa-2 cells, respectively. Western blot band densitometry intensities were determined using Image Lab 6.0 Software and normalized against GAPDH and the respective total proteins, plotted against the concentrations used. Data is representative of three independent experiments. Statistical significance (^*^p < 0.05, ^**^p < 0.01, and ^***^p < 0.001) was determined by One-way ANOVA with post hoc Dunnett’s test.

The PI3K/AKT pathway is another signaling pathway regulated by KRAS that is frequently activated in various neoplasms [26]. The effect of PCAIs on the AKT phosphorylation sites, (Ser 473) and (Thr308) was evaluated using western blotting. After 48 h treatment with 5 μM NSL-YHJ-2-27, significant 72 and 190% increases in AKT phosphorylation on Ser473 and Thr308 (Figure 4A, 4A1, 4A2) were observed in PANC-1 cells. Similarly, there were significant 97 and 82% increases in the phosphorylation of AKT in MIA PaCa-2 cells respectively at Ser473 and Thr308 (Figure 4B, 4B1, 4B2).

### PCAIs induce ROS production

**Figure 5:**
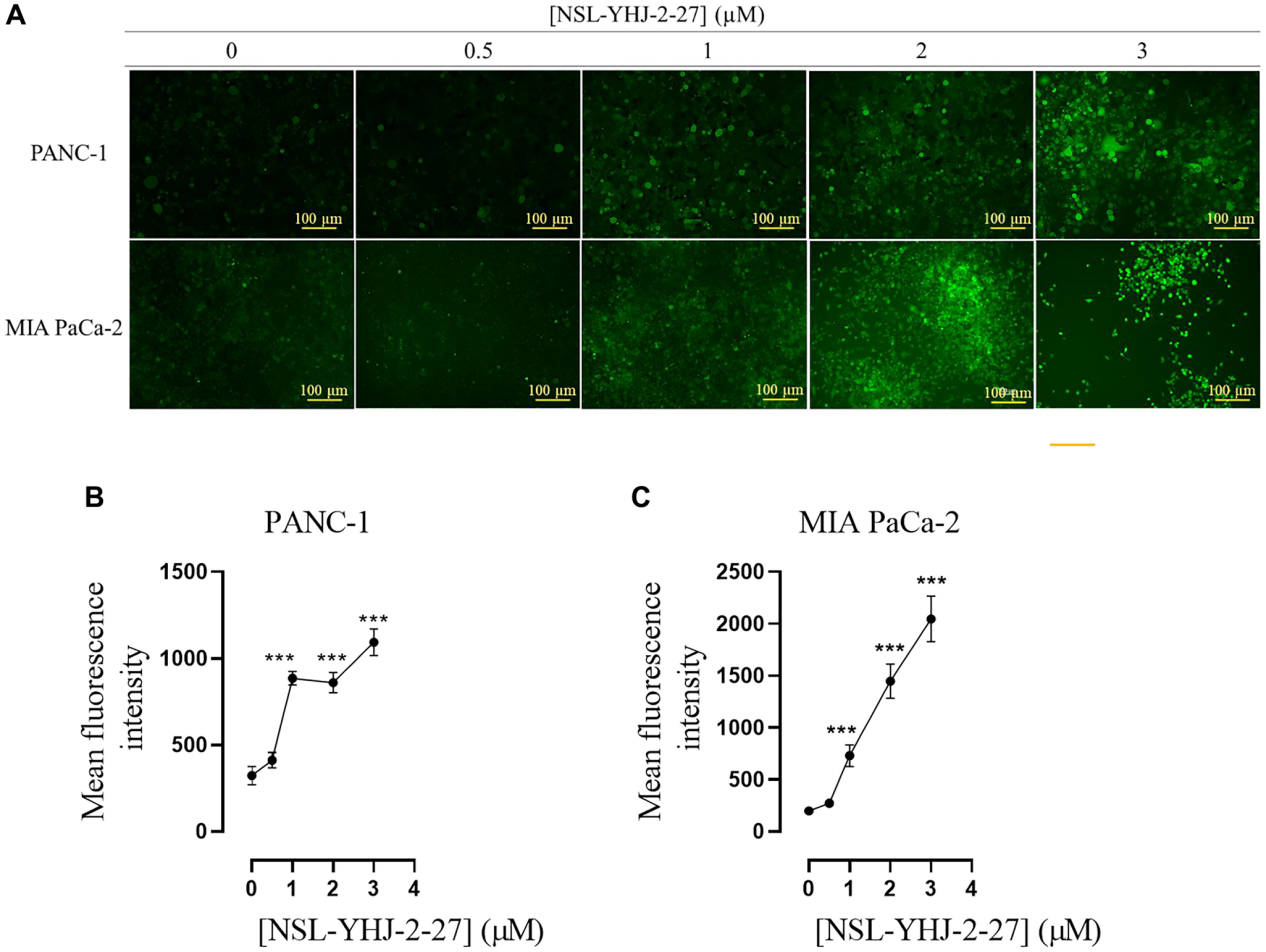
PCAIs stimulate the production of ROS in PANC-1 and MIA PaCa-2 cells. Cells were cultured in 24-well plates and treated with 0, 0.5, 1, 2, 3, and 5 μM of NSL-YHJ-2-27 for 48 h, washed with 1 X PBS and incubated with DCFH-DA working solution for 45 min. (**A**) Fluorescent images depicting ROS production in both PANC-1 and MIA PaCa-2 were captured using the Keyence BZ-X800 series microscope at 10X magnification. Quantification of mean fluorescent intensities was conducted with Keyence BZ-800 analyzer. (**B**, **C**) The mean fluorescence intensities against concentration were plotted using GraphPad Prism. One-Way ANOVA was used to determine statistical significance between the controls and samples treated with NSL-YHJ-2-27 (^***^p < 0.001). The results are representative of triplicate experiments.

AKT hyperphosphorylation has been linked to the generation of ROS [44, 45]. When these reach cytotoxic levels, damage to DNA, lipids and proteins ensues resulting in the inhibition of cell growth and apoptosis [44, 45]. Given that the PCAIs treatment results in significant phosphorylation activation of AKT, PCAIs-treated cells were probed for ROS levels using a fluorescence assay. As shown in Figure 5A, the fluorescent intensity in cells treated with PCAIs significantly increased in both MIA PaCa-2 and PANC-1 cells with increasing concentrations of PCAIs. When the fluorescence intensities were quantified, it was found that the intensities, which reflect the levels of ROS, increased by 175, 430, 240% in PANC-1 cells treated with 1, 2 and 3 μM of NSL-YHJ-2-27, respectively (Figure 5B). Similarly, MIA PaCa-2 cells treated with 1, 2 and 3 μM NSL-YHJ-2-27 showed increases of ROS as depicted by the increased fluorescence of 270, 630, 930%, respectively (Figure 5C).

### PCAIs alter MIA PaCa-2 transcriptome

**Figure 6:**
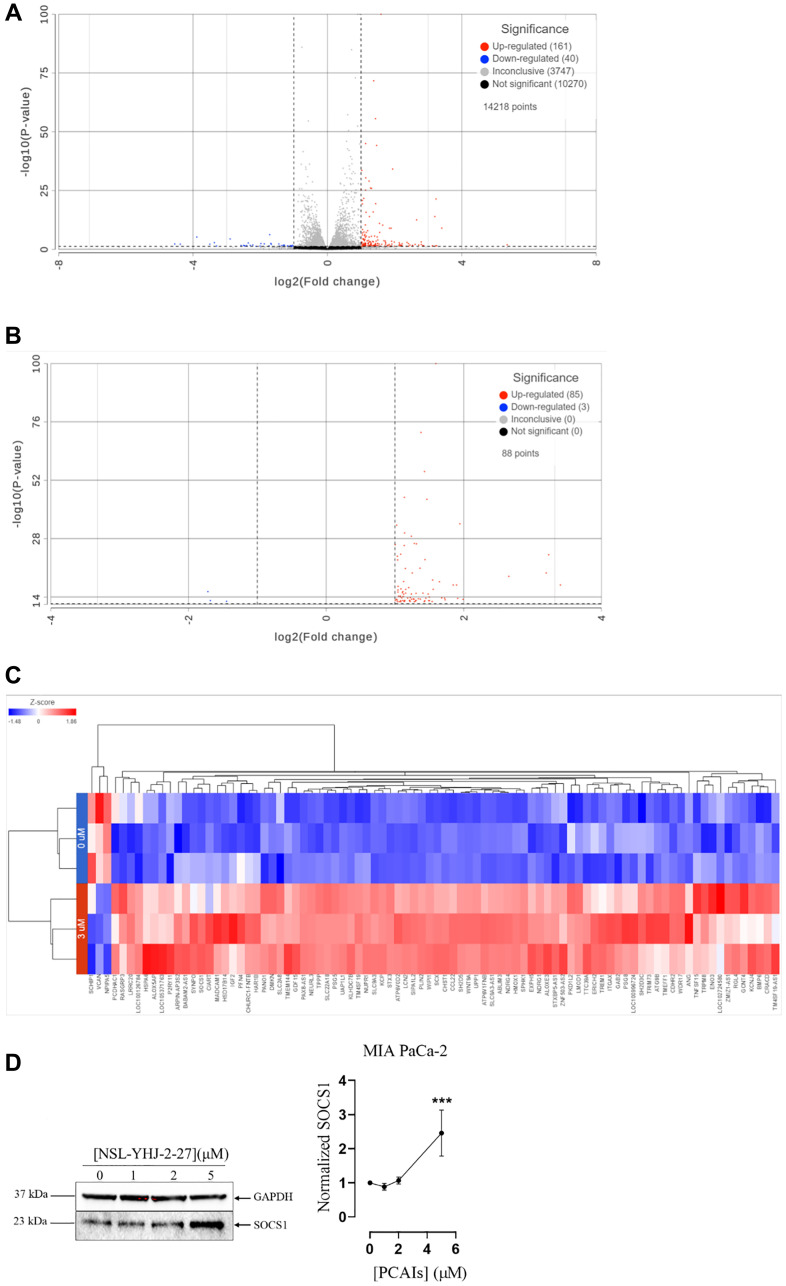
PCAIs-induced differential expression in MIA PaCa-2 cells. (**A**) After 48 h treatment with 0 and 3 μM NSL-YHJ-2-27, RNA was extracted, processed and analyzed using Partek Flow as described in the methods. DESeq2 with no cutoff threshold limits was used to distinguish gene expression between the 0 and 3 μM treatments. (**B**) DESeq2 was used to generate a list of significantly differentially expressed genes using the data set from A. False Discovery Rate (FDR) was set to include genes with *p*-values of less than or equal to 0.05 and fold-change set at log_2_ -2 and log_2_ 2 to eliminate insignificant changes in gene expression. (**C**) Hierarchical clustering was performed on the list of genes that were significantly differentially expressed to produce the heatmap. (**D**) Western blotting of SOCS1 protein to validate transcriptomic analysis.

The effects of cancer therapies are typically preceded by changes in gene expression that then culminate in the phenotypic responses to the specific therapies [46]. Differential expression analysis aims to identify these changes to better understand the mechanisms of action and identify more opportunities for therapeutic improvements. Here, we determined the effect PCAIs on mRNA transcripts in MIA PaCa-2 cells. After 48 h treatment with 3 μM NSL-YHJ-2-27, differential gene expression using DESeq2 from Partek Flow software (Sacramento, CA, USA) revealed a total of 201 affected genes. Approximately 161 genes were upregulated, and 40 genes were downregulated (Figure 6A). After performing DESeq2 analysis using cutoff thresholds to include genes with adjusted *p*-value of less than or equal to 0.05 and fold change of -2 and lower and 2 and higher, 85 genes were significantly upregulated, and 3 genes were found to be significantly downregulated (Figure 6B). The 88 genes whose expression levels were significantly altered were visualized using hierarchical clustering to produce a heatmap (Figure 6C). Genes that implicate the role of PCAIs in impeding PDAC progression such as heme oxygenase-1 (*HMOX1*) and autophagy-related protein 9 (*ATG9B*) were upregulated and Schwannomin-interacting Protein 1 (*SCHIP1*) and Versican (*VCAN*) were downregulated. We validated the PCAIs-induced effects on gene expression by demonstrating the 150% increase in the levels of suppressor of cytokine signaling 1 (*SOCS1*) (Figure 6D).

### PCAIs inhibit pancreatic cancer cell migration and 3D spheroid invasion

**Figure 7:**
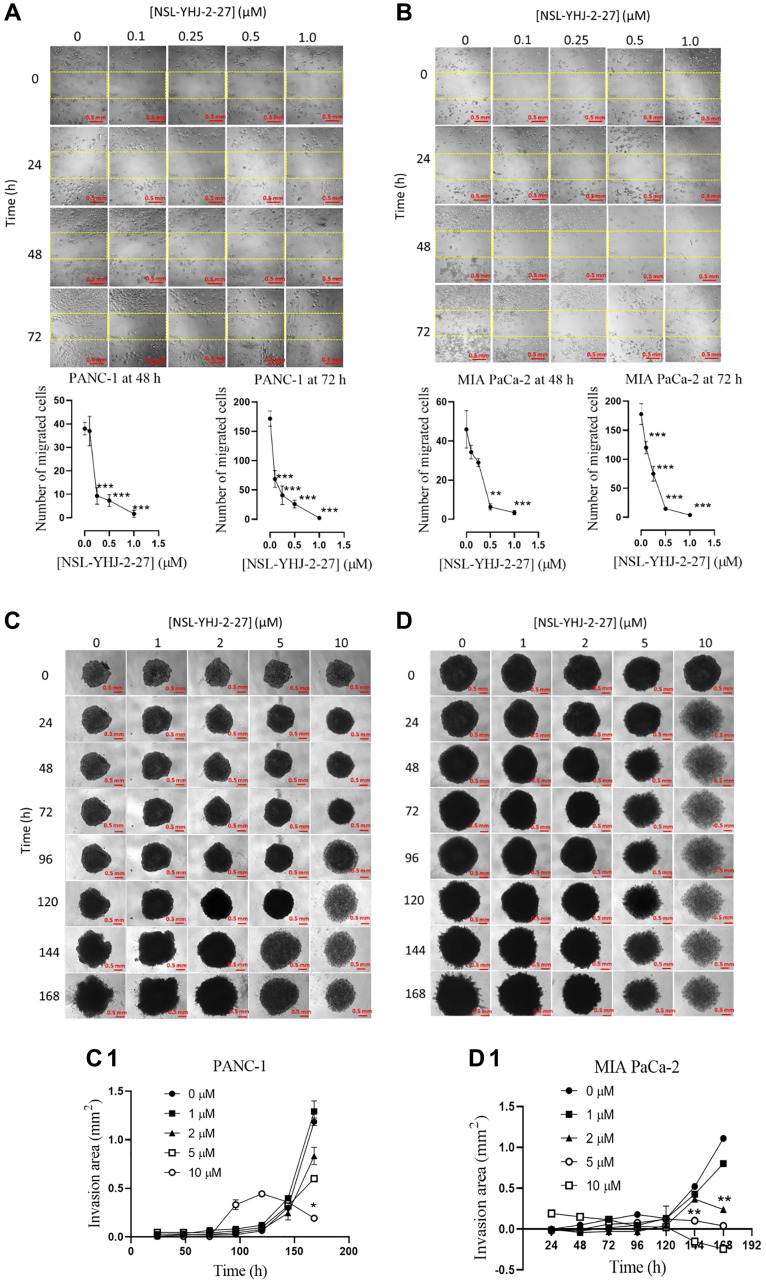
PCAIs inhibit the migration and 3D spheroid invasion of pancreatic cancer cells. (**A**, **B**) Monolayers of PANC-1 and MIA PaCa-2 cells separated by a “wound” were treated with the indicated concentrations of NSL-YHJ-2-27. Closure of the wound was monitored and images captured at 0, 24, 48 and 72 h after treatment using the Nikon Ti Eclipse microscope at 10X magnification. The number of cells that migrated into the standard “wound” areas were counted. Data of nine images per concentration and time point were analyzed by One-Way ANOVA with Dunnett’s posthoc test, statistical significance (^*^p < 0.05, ^**^p < 0.01, ^***^p < 0.001). (**C**, **D**) PANC-1 and MIA PaCa-2 preformed spheroids in experimental media were treated with the indicated concentrations of NSL-YHJ-2-27. The spheroids were then submerged in PCAIs-treated Matrigel and kept for 30 min to solidify. Images were captured at the onset and every 24 h thereafter for seven days using a Nikon Ti Eclipse microscope at 4X magnification. Time-dependent changes in spheroid invasion areas were measured for each treatment concentration and quantified using NIS-Elements AR version 4.30. (**C1**, **D1**) Invasion areas were plotted against PCAIs concentration. Two-Way ANOVA with Dunnett’s posthoc test was used to determine statistical significance (^*^p < 0.05, ^**^p < 0.01).

Metastasis is the major cause of malignancy especially for pancreatic cancer that is characterized by high mortality rates [47]. Since metastasis involves cell migration and invasion of other tissues to initiate secondary tumors, we therefore determined the effect of the PCAIs on cell migration using the “wound” healing assay. The results show significant decreases in the number of migrated cells into the “wound” by PANC-1 cells whereby 0.1, 0.25 and 0.5 μM NSL-YHJ-2-27 inhibited the migration by 60, 76 and 85%, respectively (Figure 7A). Similar results were obtained in MIA PaCa-2 whereby 0.1, 0.25 and 0.5 μM NSL-YHJ-2-27 inhibited migration by 33, 58 and 92% respectively, after 72 h exposure (Figure 7B). A large proportion of the cells in both cell lines did not survive treatment with 1 μM NSL-YHJ-27. Invasion into Matrigel by PANC-1 and MIA-PaCa-1 spheroids was significantly inhibited by 84% and 96%, respectively in the presence of 10 μM NSL-YHJ-2-27 (Figure 7C, 7C1, 7D, 7D1). PANC-1 and MIA PaCa-2 spheroid disaggregation was visible within 96 and 24 h of treatment with 10 μM NSL-YHJ-2-27, respectively. The disaggregation resulted in an expanded and grayish appearance of the spheroids, hence the parabolic nature of 10 μM graph line (Figure 7C1).

### PCAIs suppress actin filaments in pancreatic cancer cells

**Figure 8:**
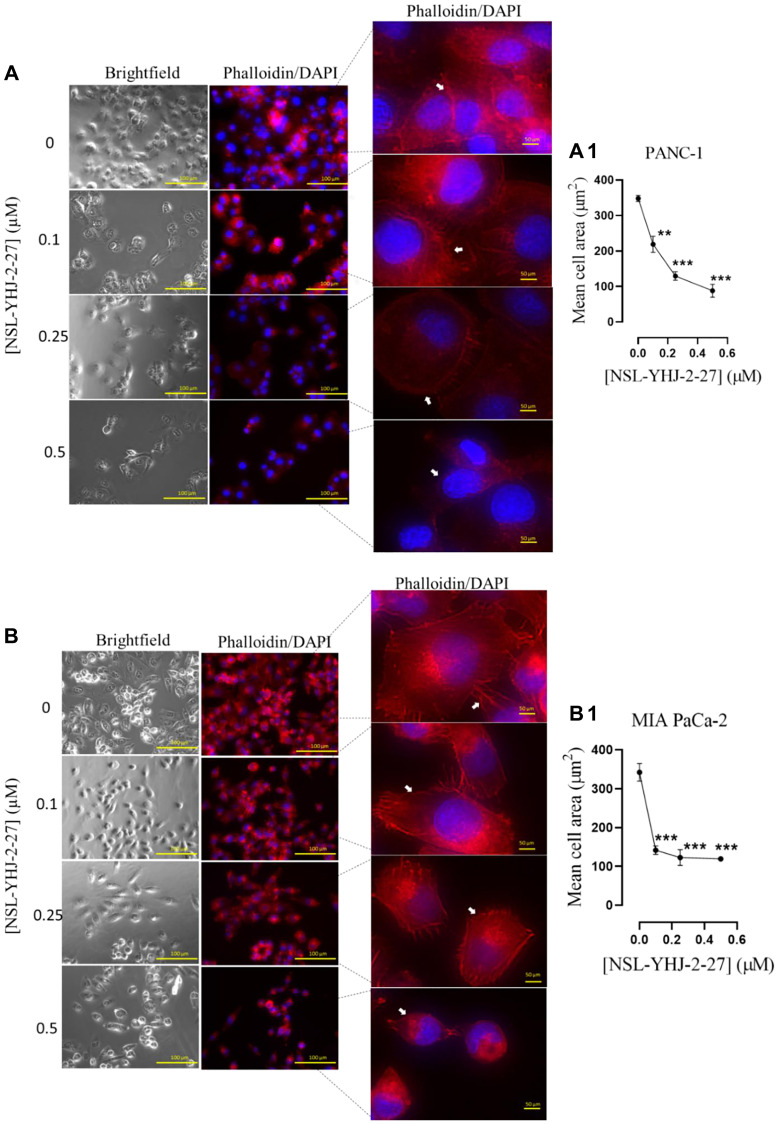
PCAIs disrupt F-actin filaments in pancreatic cancer cells. Monolayers of (**A**) PANC-1 and (**B**) MIA PaCa-2 cells were seeded onto an 8-well μ slide plate (ibidi) and treated with the indicated concentrations of PCAIs for 48 h. The treated cells were then fixed and stained with Alexa Fluor™ 568 Phalloidin mixed with Hoechst reagent. Fluorescent images of the F-actin cytoskeleton were captured with the Keyence BZ-X800 series microscope at 40X magnification. The white arrows point to the F-actin filaments. (**A1**, **B1**) The mean cell areas were quantified using the BZ-800 analyzer and plotted against PCAIs concentration. Statistical significance (^*^p < 0.05, ^**^p < 0.01, and ^***^p < 0.001) was determined by One-way ANOVA for PANC-1 and MIA PaCa-2 cells with post hoc Dunnett’s test.

Actin polymerization provides the cytoskeletal framework essential for cell shape, motility and consequently cell migration and invasion which are contributing hallmarks for metastasis and angiogenesis [35]. In cancer cells, these structures are dysregulated resulting in excessive cell motility resulting in metastasis [35, 48]. Disruption of these cytoskeletal structures leads to altered cell morphology, diminished cell compactness and decreased cell migration. We determined the effects of PCAIs on the F-actin filaments in PANC-1 and MIA PaCa-2 cells using Alexa Fluor™ 568 Phalloidin mixed with Hoechst stain (Figure 8). At 0.5 μM, NSL-YHJ-2-27 caused cell rounding and collapse of the F-actin filaments, resulting in significant 75% and 65% decreases, respectively, in mean PANC-1 (Figure 8A, 8A1) and MIA PaCa-2 cell areas (Figure 8B, 8B1). The decreases in cell area were associated with corresponding increases in the spaces between the treated cells compared to the controls, due to the retraction of actin filament-based structures, such as lamellipodia and filopodia.

### PCAIs induce apoptosis in 2D monolayer cells and 3D spheroids

**Figure 9:**
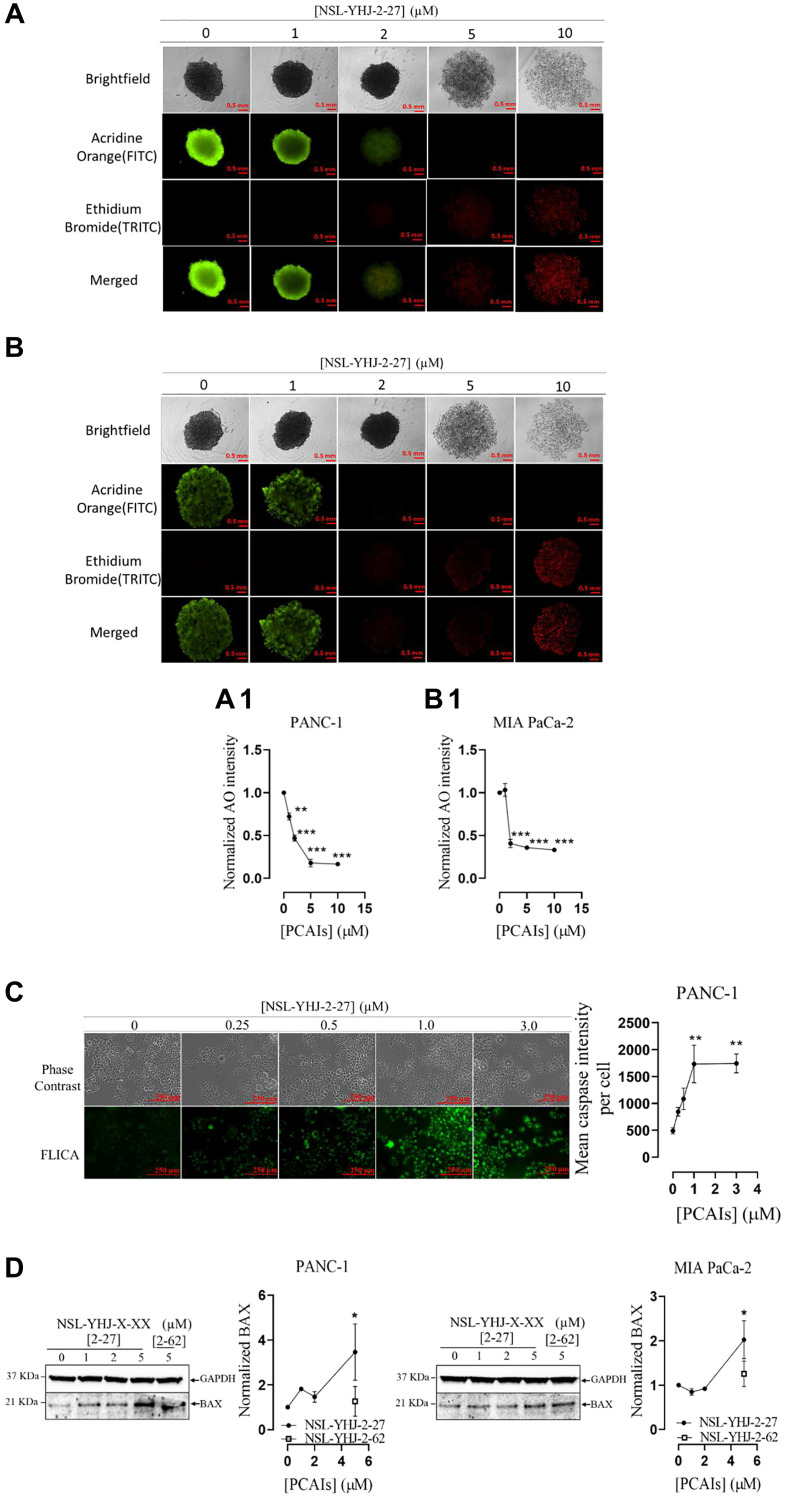
PCAIs induce apoptosis in PANC-1 and MIA PaCa-2 cells. (**A**, **B**) PANC-1 and MIA PaCa-2 spheroids were treated with the indicated concentrations of PCAIs for 48 h. At 96 h, the spheroids were stained with AO/EB (5 μL, 100 μg/mL) and immediately imaged using the Nikon Ti Eclipse microscope. The fluorescence intensities of the captured images were determined, and the normalized mean AO fluorescence intensities were plotted against concentration (**A1** and **B1**). One-Way ANOVA with Dunnett’s posthoc test was used to determine statistical significance (^**^p < 0.01, ^***^p < 0.001) (**C**) PANC-1 cells were treated with the indicated concentrations of NSL-YHJ-2-27 in 8-well μ slide plate (ibidi) for 48 h. This was followed by treatment with the CaspaTag™ specific active caspase-3/7 labeling reagent. The fluorescence intensities were measured using the Keyence BZ-X800 fluorescence microscope and quantified using the Keyence BZ-X800 analyzer software. Mean fluorescence intensities per cell were plotted against the respective NSL-YHJ-2-27 concentrations. Statistical significance (^**^p < 0.01) was determined by One-Way ANOVA with post hoc Dunnett’s test. (**D**) Western blotting was used to determine the levels of BAX protein in NSL-YHJ-2-27-treated PANC-1 and MIAPaCa-2 cells. The results are representative of three independent experiments. Statistical significance (^*^p < 0.05) was determined by one-way ANOVA for PANC-1 and MIA PaCa-2 cells with post hoc Dunnett’s test.

Tumors are three dimensional masses of proliferating cells characterized by compactness and microenvironments that constitute crucial determinants of their responsiveness to various therapies [47]. The 3D nature of tumors was replicated for PANC-1 and MIA PaCa-2 cells by developing 3D spheroids *in vitro* to investigate the effect of PCAIs. Treatment of PANC-1 spheroids with NSL-YHJ-2-27 at 5 and 10 μM decreased the proportion of live cells by 79 and 74%, respectively (Figure 9A, 9A1). Similar results were obtained for MIA PaCa-2 spheroids whereby number of live cells decreased by 63 and 62%, respectively (Figure 9B, 9B1). At 2 μM NSL-YHJ-2-7, equal amounts of live to apoptotic cells were observed for both cell lines. At 5 μM, we observed disintegration and collapse of the spheroids. Only dead cells of both PANC-1 and MIA PaCa-2 spheroids remained. Furthermore, when the cells were treated with the PCAIs for 48 h and probed with the CaspaTag™ Caspase-3,7 *in situ* reagent, fluorescence intensities increased with increasing PCAIs concentrations. At 3 μM NSL-YHJ-27, the active caspase levels as denoted by the fluorescence intensities were 260% higher than in control untreated cells (Figure 9C). Furthermore, Western blotting revealed that the levels of the proapoptotic protein BAX significantly increased in PANC-1 and MIA PaCa-2 by 246 and 95% at 5 μM NSL-YHJ-2-27, respectively (Figure 9D).

### Overall effects of PCAIs on PDAC cancer cells

**Figure 10:**
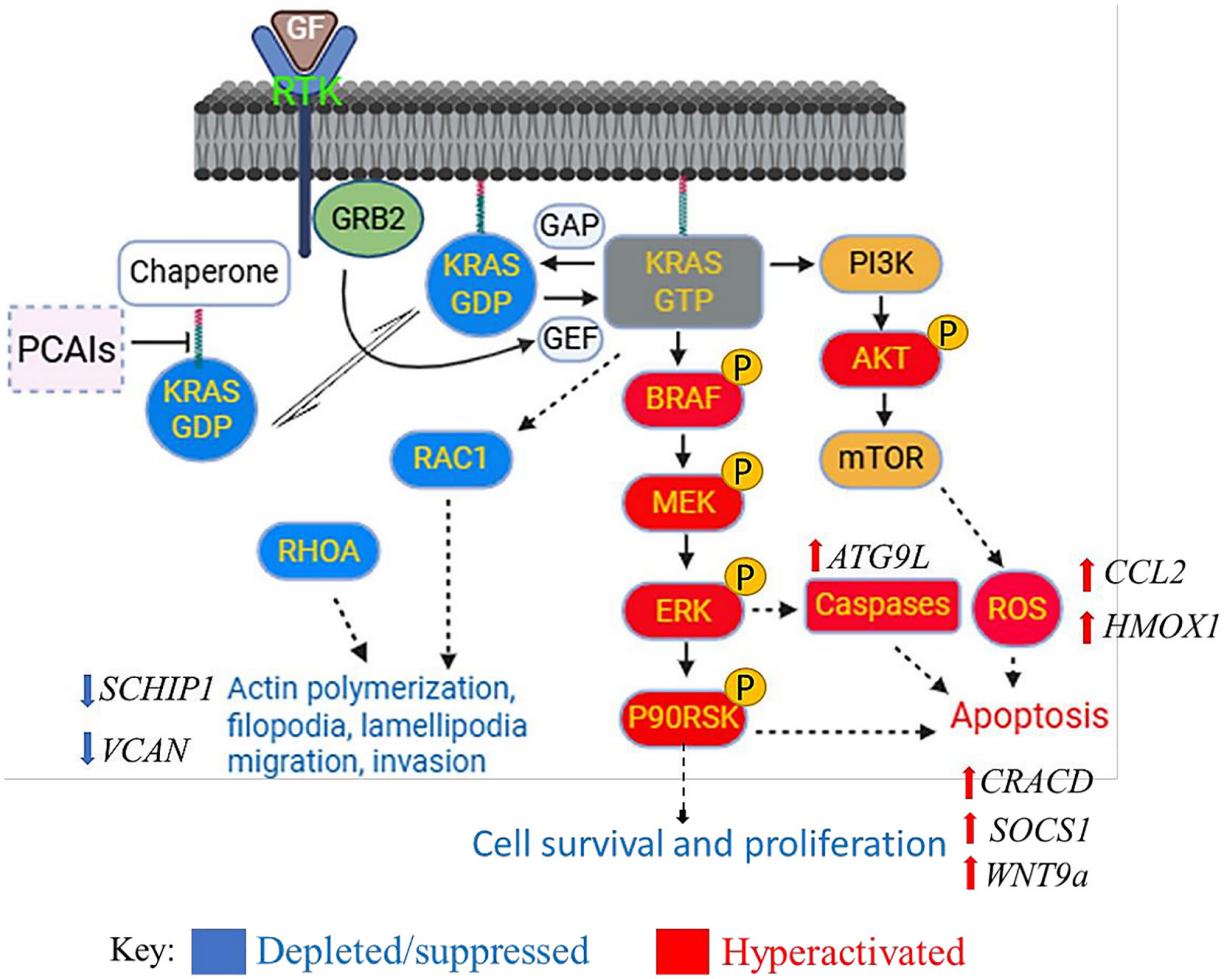
Biochemical effects of PCAIs on cancer cells. PCAIs-treated cancer cells show the depletion of the monomeric G-proteins RAC1 and RHOA. MAPK and PI3K/AKT signaling pathways are activated resulting in the generation of ROS, caspases activation and apoptosis. Actin filaments and its dependent processes were also suppressed resulting in cell rounding and possibly anoikis. Key genes that impact cell survival and cancer progression were either upregulated or downregulated upon PCAIs treatment.

The overall effects of PCAIs treatment on the cancer cell are summarized in Figure 10. While the PCAIs had no significant effect on the levels of KRAS, RAC1 and RHOA levels were significantly reduced. The PCAIs induced the hyperphosphorylation of BRAF, MEK, ERK, and P90RSK of the MAPK pathway and AKT of the PI3K/AKT pathway. Decreased proliferation was observed in conjunction with the generation of ROS and caspases ultimately resulting in apoptosis. PCAIs also disrupted actin filaments and suppressed their dependent processes, resulting in cell rounding and possibly anoikis. Key genes that suppress cancer progression processes, such as *CRACD, ATG9L, SOCS1, HMOX1, WNT9a*, and CCL2 were upregulated while cancer promoting genes such *as VCAN* and *SCHIP1 were downregulated*.

## DISCUSSION

The decades long search for drugs targeting oncogenic KRAS-driven cancers has only recently been met with success thanks to the introduction of the KRAS^G12C^-targeting agents, Sotorasib and Adagrasib [49, 50]. In addition to the limitation of targeting only one of several oncogenic mutant forms of KRAS, Sotorasib tends to lose efficacy due to intrinsic tumor cell resistance after only about 18 months [31, 51]. Consequently, novel therapies are needed to help manage pancreatic cancers driven by other mutant KRAS proteins as well as those that have become resistant to KRAS^G12C^-targeting drugs. One class of such promising agents is the PCAIs that were designed to target oncogenic G-proteins in a manner that is different from the KRAS^G12C^-targeting drugs. Designed to mimic the C-terminal post-translational modifications of G-proteins, PCAIs are effective against a broader range of KRAS mutants, including KRAS^G12C^, KRAS^G12D^, and KRAS^G12V^, thereby indicating potential pan-KRAS anticancer applicability. Indeed, our previous works revealed the effectiveness of the PCAIs against breast, lung and prostate cancer cells driven by mutant KRAS proteins [19, 35, 52–54]. In the current work, we determined that PCAIs effectively inhibit the viability of PANC-1 cells. To substantiate the belief that the PCAIs target the polyisoprenylation-dependent interactions of G-proteins for disruption, PCAIs with the farnesyl group are always potent against various cancer-promoting phenomena compared to the largely ineffective compounds NSL-YHJ-2-62, NSL-YHJ-2-31 and NSL-YHJ-56 that lack it. This is to be expected as the polyisoprenyl modifications of RAS proteins facilitate their protein-protein interactions and functional localization [13, 17, 55].

Mutant KRAS signals through the MAPK and AKT pathways that leads to cancer cell characteristics such as tumorigenesis, metastasis and excessive cell growth [21]. Based on the observation that the PCAIs induce cell death, we anticipated that they suppress the signaling of the kinases downstream of RAS, since PCAIs mechanism of action may be due to the uncoupling of mutant KRAS-proteins from interactions with other effectors. On the contrary, we observed phosphorylation hyperactivation of MAPK proteins BRAF, MEK1/2, ERK 1/2 and P90RSK, and AKT. Although these findings were initially unexpected, hyperactivation of certain MAPK proteins have been demonstrated to be associated with apoptosis. For instance, the hyperactivation of ERK 1/2 has been linked to induction of proapoptotic BH3 proteins [56, 57]. The significant increase in BH3 protein BAX, as well as the induction of caspases 3/7 activity and the observed cell death in the spheroids further substantiate that NSL-YHJ-2-27 induces apoptosis. The P90RSK protein has four isoforms, P90RSK 1 and 2 promote cell proliferation while activation of P90RSK 3 and 4 result in cell death [58, 59]. Also, the activation of MAPK and AKT pathways by the PCAIs resulting in cancer cell death [35, 52] is consistent with the upregulation of the Ras guanyl nucleotide releasing peptide 3 (*RASGRP3*) gene that regulates RAS downstream signaling [60]. Although RASGRP3 overexpression has been linked to tumor progression, some reports show that overstimulation of RAS due to RASGRP3 overexpression leads to hyperphosphorylation of MAPK signaling pathway enzymes in cancer cells, disrupting homeostasis that results in cell death [44, 45, 61, 62]. Das Thakur and co-workers demonstrated that overstimulation of MAPK in 45V-RT melanoma cells resulted in increased ROS generation and cell death [62].

The observations that MAPK and PI3K/AKT pathways activation results in PDAC cell death is consistent with our previous observations of cell death following PCAIs-induced MAPK and PI3K/AKT [35] pathways activation in lung and breast cancer cell lines [44, 56]. Although activation of these pathways typically promotes cell growth and survival, cell death following AKT activation has been reported [44, 45]. PI3K/AKT pathway proteins have both cytoplasmic and nuclear localizations where they are believed to impact different cadres of substrates [44]. In the nucleus, AKT interactions with antiapoptotic factors promotes apoptosis [44]. AKT phosphorylation also suppresses the expression of antioxidant enzymes, which results in accumulation of ROS leading to cell death [45]. Both mechanisms appear to have played a role in the observed cell death as proapoptotic factors and ROS were both detected in the dying cells. Also, it was previously reported that the disruption chaperone-KRAS trafficking may cause hyperphosphorylation of p-AKT, that may then induce ROS production. [35, 52]. Moreover, the generation of ROS by PCAIs is possibly due to the observed upregulation of C-C motif chemokine ligand 22 (*CCL22*), a gene associated with oxidative stress. This may imply that the PCAIs induce transcriptional changes resulting in the increased production of ROS [63, 64]. With CCL22 being a chemoattractant, tumor-associated macrophages (TAMs) M1 and M2 which have anti- and pro-tumor effects, respectively, are recruited and these TAMs may then infiltrate the tumor resulting in tumor death [64–66]. Additionally, heme oxygenase-1 (*HMOX1*) gene which acts as a crucial antioxidant in response to cellular stress by scavenging ROS [67] was overexpressed. These transcriptional changes are possibly defense mechanisms of MIA PaCa-2 cells in response to the cytotoxic levels of ROS produced by PCAIs treatment [63, 67]. However, it is important to note that the hyperstimulation of both the MAPK and AKT pathways may not be due to direct interaction with the PCAIs. The hyperphosphorylation of enzymes in the MAPK and AKT pathways may be due to PCAIs disruption of the interactions of G-protein such as KRAS. This is understandable as PCAIs are structurally similar to and likely compete with their polyisoprenylation-dependent protein complexes resulting in altered protein-protein interactions and signaling patterns. For example, it has been widely reported that G-proteins such as KRAS are bound to and are trafficked by calmodulin (CALM) [14, 16] in an interaction that is strongly dependent on polyisoprenylated C-terminal hypervariable region [17].

Metastasis and invasion are major debilitating characteristics of tumor cells that cause high morbidity and mortality rates amongst cancer patients [68]. Therefore, therapies that abrogate tumor cell motility have a strong potential to positively impact cancer management. Monomeric G-proteins such as RAC1, CDC 42 and RHOA which play key roles in tumor cell motility become hyperactivated in cancer. These have been implicated in the cancer cell invasion caused by the carcinoma-associated fibroblasts alteration of the tumor microenvironment [68–71]. Since the PCAIs are designed to mimic G-proteins that have been modified with a single polyisoprenyl moiety, their effects are expected to impact not only hyperactive KRAS but also the functions of other monomeric G-proteins such as CDC42, RHOA and RAC1 [72]. The significant decrease in RAC1 and RHOA levels in PANC-1 after PCAIs treatment may explain the inhibition of the observed cell migration and invasion. Although there were no significant changes in the levels of RAC1 and RHOA in MIA PaCa-2, the observed inhibition of cell migration could be due to the PCAIs alternative mechanisms such as interference with focal adhesion proteins such as vinculin and fascin that promote cytoskeletal stability and cell movement [72]. Our previous work revealed the depletion of vinculin and fascin in lung and breast cancer cells after treatment with micromolar concentrations of PCAIs [48, 52]. Further effects that negatively impact cell migration and invasion and consequently metastasis and angiogenesis include the cell rounding and decrease in cell volume. These were previously observed in lung cancer cells where decreases in filamentous actin, loss of filopodia and lamellipodia resulted in the loss of cell shape and cell migration [48, 73]. The effects against cancer cell migration and invasiveness is further corroborated by the upregulation of capping protein inhibiting regulator of actin dynamics (CRACD), involved in negative regulation of barbed-end actin filament capping [74]. Inactivation of CRACD has been linked to deregulation of F-actin polymerization resulting in increased cell migration [74, 75]. CRACD also acts as a tumor suppressor, that has been reported to restrict plasticity in A549 lung adenocarcinoma cells [76, 77].

From the phenotypic responses observed after NSL-YHJ-2-27 treatment, we desired to know any possible changes at the gene expression level. The PCAIs-induced downregulation of Schwannomin-interacting Protein 1 (*SCHIP1*) gene, a cytoskeletal-associated protein that interacts with the focal adhesion molecule ankyrin for mechanical support to the plasma membrane [78] explains, at least in part, the observed spheroid disaggregation of PANC-1 and MIA PaCa-2. Previous experiments revealed that PCAIs inhibit not only cell proliferation and viability in lung, breast and prostate cancer cells [52, 54, 72] but also tube formation and angiogenesis in HUVEC cells and chick embryos [79]. The observed downregulation of Versican (*VCAN*) genes that play critical roles in cell adhesion and angiogenesis partly explains the PCAIs inhibition of angiogenesis [80]. Studies indicate that VCAN is often overexpressed in several cancers such as brain, breast, gastric, and renal [81–83], and the absence of VCAN enhances the efficacy of drugs such as cisplatin, gemcitabine and epirubicin against upper urinary tract urothelial carcinoma [82]. Additionally, MIA PaCa-2 is homozygous for the *KRAS^G12C^* mutation [84, 85] while PANC-1 is heterozygote for *KRAS^G12D^* mutation [84, 85], differences that may explain the disparate biological responses to PCAIs treatment. Cell lines with homozygote mutations show slightly more aggressive cancer characteristics such as cell proliferation and metastasis compared to their heterozygote counterparts [85]. The NSL-YHJ-2-27 effects on both cancer cells possessing different KRAS mutations highlight the broader potential applicability of the PCAIs to treat cancers driven by different mutant KRAS forms than drugs targeting only specific mutants. After PCAIs treatment we observed significantly increased expression of the suppressor of cytokine signaling 1 (*SOCS1*) gene. PCAIs ability to inhibit the viability of MIA PaCa-2 cells could stem from the observed upregulation of the *SOCS1*, which prevents T-cell degradation and impedes cell proliferation in cancer cells [86]. SOCS1 is a tumor suppressor which negatively regulates the JAK/STAT pathway and is often silenced in tumor cells [84–88]. Our observation is further substantiated using western blotting showing the PCAIs-induced overexpression of SOCS1. Another notable gene that was upregulated is the *WNT9a* gene that expresses WNT9a, a protein involved in Wnt signaling [89]. The *WNT9a* is considered a tumor suppressor whose expression is often suppressed in gastric, breast and pancreatic cancers [89]. Ali et al, induced WNT9a expression using LiCl which suppressed colorectal cancer (CRC) cells [90]. Also, autophagy-like proteins such as ATG9 and ATG12 play vital roles during the early stages of tumorigenesis, promoting the formation of autophagosomes, structures that engulf damaged cellular components for breakdown, preventing tumor formation and suppressing cancer growth [91, 92]. The PCAIs ability to inhibit the viability of PANC-1 and MIA PaCa-2 cells could also be attributed to the 3-fold upregulation of autophagy-related protein 9 (*ATG9L*) gene [92]. The PCAIs-induced upregulation of genes such as the neutralized E3 ubiquitin protein ligase 3 (*NEURL3*) and solute carrier family 22 member 18 (*SLC22A18*) possibly led to MIA PaCa-2 cell death through ubiquitination, since both genes are directly involved in ubiquitin ligase activity [93, 94]. Finally, we observed that approximately 87 of the 88 genes that were differentially expressed after PCAIs treatment are not involved with general toxicity except for the solute carrier family 9-member A3 (*SLC9A3*) whose overexpression has been linked with congenital secretory sodium diarrhea [95]. Planned *in vitro* and *in vivo* studies will be conducted to assess the PCAIs as potential pan-mutant KRAS inhibitors.

## MATERIALS AND METHODS

### Materials

The PCAIs, NSL-YHJ-2-27, NSL-YHJ-2-45 and NSL-YHJ-2-62, were synthesized in our lab as previously described [19]. The human pancreatic adenocarcinoma cell lines, MIA PaCa-2 and PANC-1 were purchased from ATCC, Manassas, VA. Dulbecco’s Modified Eagle’s medium and phosphate buffered saline (PBS) were purchased from Invitrogen, Waltham, MA, fetal bovine serum (FBS), penicillin and streptomycin were obtained from Atlanta Biologicals, Atlanta, GA, USA. CaspaTag™ Caspase-3,7 *In Situ* Assay Kit (Cat# APT403), and 2,7 Dichlorofluorescein Diacetate (DCFH-DA, Cat# D6883, reagent grade) were obtained from Sigma-Aldrich (St. Louis, MO, USA). Primary monoclonal antibodies for mitogen activated protein (MAP) kinase and AKT/PI3K pathways; total MEK 1/2 (Cat #8727S), phosphorylated MEK 1/2 (p-MEK 1/2, Cat #9154), total ERK 1/2 (Cat#4695S), phosphorylated ERK 1/2 (p-ERK1/2, Cat #4370), total P90RSK (Cat #9355S), total AKT (Cat #4691S), phosphorylated AKT (p-AKT(Ser473, Cat#4060S), phosphorylated P90RSK (p-P90RSK, Cat #11989), RAC1/2/3 (Cat #2465), RHOA (Cat #2117S) and BH3-only monoclonal antibody, BAX (Cat # 5023S) were purchased from Sigma-Aldrich, St. Louis, MO, USA.

### Cell culture and treatment

MIA PaCa-2 and PANC-1 cells were cultured at 37°C in T-75 flasks in DMEM growth media supplemented with 10% FBS, 1% penicillin and streptomycin with 5% CO_2_, 95% humidified air. The cells were sub-cultured when at 80–90% confluency. Unless stated otherwise, experiments using the cells were conducted in experimental media containing 5% FBS. The concentrations used in the current study were based on effective concentration/EC_50_ values against cell viability studies and from this and previous experiments [19, 20, 35, 48].

### Effect of PCAIs on cell viability

MIA PaCa-2 and PANC-1 cells were seeded at 10,000 cells/per well into 96-well plates with 100 μL of 5% FBS-supplemented experimental media and treated with PCAIs (contained in 1 μL of acetone) to final concentrations from 0–50 μM. These treatments were repeated after 24 h. Equivalent 1 μL volumes of acetone were used to treat control cells. At 48 h from the onset of treatments, 20 μL of 0.02% of reagent grade resazurin dye (Beantown Chemicals, Sangamare, NH) was added into each well and incubated for 3 h to determine the effect of PCAIs on cell viability. After 3 h incubation, fluorescence measurements were conducted at excitation and emission wavelengths of 540 nm and 590 nm, respectively, using the FLx 800 Microplate Fluorescence Reader from BioTek. The EC_50_ values were extrapolated from non-linear regression plots of the logs of the PCAIs concentrations against cell viability using Graph Pad Prism software.

### Effect of PCAIs on phosphorylation of MAPK and PI3K/AKT pathway proteins

The two pancreatic cancer cell lines MIA PaCa-2 and PANC-1 cells were each seeded at 1.5 million cells per well into 60.8 cm^2^ culture dishes containing 10 mL of experimental media supplemented with 5% FBS. To determine the effect of the PCAIs on the MAPK and PI3K pathway proteins, the plated cells were treated with 20 μL of the PCAIs, NSL-YHJ-27 to final concentrations of 0 (acetone/carrier solvent at 0.2% final concentration for controls), 1, 2 and 5 μM. These treatments were repeated after 24 h. At 48 h, the cells were rinsed with 1X PBS and lysed with 200 μL of lysis buffer (1% PBS, 0.1% triton X-100) supplemented with 0.1% v/v protease/phosphatase inhibitors cocktail (Cell Signaling Technology, Danvers, MA, USA). The protein concentrations of the lysates were determined using the Bradford assay (Quick Start). Cell lysates containing 30 μg of protein were combined with 10 μL of 20x XT reducing agent (BioRad, Hercules, CA, USA) and 50 μL of 4x XT sample buffer and boiled for 5 min. Following SDSPAGE separation on 4–12% Criterion™ XT Bis-Tris protein gels, the proteins were transferred onto Trans-Blot turbo midi 0.2 μm nitrocellulose membranes (Bio-Rad, Hercules, CA, USA). OneBlock™ western-CL blocking buffer (Genesee Scientific, San Diego, CA, USA) was used to block the membranes for 1 h at room temperature. The blocked membranes were then incubated overnight at 4°C in fresh blocking buffer containing the corresponding primary monoclonal antibodies. The membranes were then washed three to five times with 5 to 10 mL of 1x Tris buffer saline Tween-20 (TBST) and incubated with HRP-linked anti-rabbit or anti-mouse antibodies for 1 h at room temperature. The ChemiDoc XRS+ System (Bio-Rad, Hercules, CA, USA) was used to visualize immunoreactive bands using Radiance Plus (Azure Biosystems, Dublin, CA, USA) ECL reagent in accordance with the manufacturer’s instructions. Total protein or protein phosphorylation levels were measured using Image Lab 6.0 (Bio-Rad, Hercules, CA, USA) and normalized against the appropriate GAPDH and/or total protein band chemiluminescence intensities. The results from three independent experiments were plotted using GraphPad Prism software version 8.0 for Windows (San Diego, CA, USA).

### Effect of PCAIs on reactive oxygen species (ROS) levels

MIA PaCa-2 and PANC-1 cells were seeded at densities of 1 × 10^5^ cells/per well of a 24 well plate containing with each well containing 500 μL of experimental medium. The cells were treated with NSL-YHJ-2-27 (0, 0.5, 1, 3 and 5 μM) for 48 h. The media were removed, and the cells washed once with serum free medium. A working solution of 25 μM 2,7 DCFH-DA was prepared using prewarmed experimental medium. Approximately 500 μL of DCFH-DA working solution was pipetted into each well and incubated for 40 min after which it was removed and the cells washed twice with experimental medium and finally once with 1X PBS. Representative fluorescent images were taken for each treatment at 10X magnification using the Keyence BZ-X800 series microscope with excitation and emission wavelengths set at 485 nm and 530 nm, respectively. The fluorescence intensities, indicative of ROS levels, were determined using the Keyence BZ-X800 analyzer and data plotted using GraphPad Prism. One-Way ANOVA was used to determine the statistical significance between PCAIs concentrations and the controls.

### Effect of PCAIs on gene expression

MIA PaCa-2 cells (3 × 10^6^ cells in 10 mL of experimental medium containing 5% FBS) were treated in triplicates with the carrier solvent, acetone (0.2% final concentration, controls) or NSL-YHJ-2-27 in acetone (3 μM) for 48 h. After treatment, the cells were harvested, prepared using directions from Novogene and sent for total RNA sequencing at Novogene, Sacramento, CA, USA. Bulk RNA processing and analysis was done using Partek Flow (St. Louis, MO, USA) as follows:

Alignment: Total unaligned mRNA data was imported into Partek Flow and the quality of the unaligned reads was assessed by pre-alignment QA/QC analysis task. The sequencing quality of unaligned reads was provided as a Phred score. The average Phred score was adequate, hence, no trimming was performed. STAR 2.7.8a index aligner was used to align mRNA reads to the whole genome with *homo-sapiens* hg19 selected for assembly. After alignment, post-alignment QA/QC was performed to determine the quality of alignment of mRNA reads with whole genome data.

Differential expression analysis: Partek E/M was used to quantify transcripts using RefSeq Transcripts 95 as the annotation model. Low expression genes were excluded by filtering out gene expressions less than or equal to 10 reads from a transcript and the total number of reads were normalized using the median ratio normalization method. The differential expression analysis tool, DESeq2 for bulk RNA-seq data was used to compare the differential expression of genes between the 0 and 3 μM NSL-YHJ-2-27 treatments. The list of differentially expressed genes for each treatment group of samples was visualized using the volcano plot and heatmap.

Enrichment analysis: KEGG pathway enrichment analysis was performed on the down- and upregulated genes to identify the pathways to which the affected genes belong.

### Effect of PCAIs on cell migration

To determine the effect of PCAIs on cell migration, the wound healing method was used. A “wound” was created in wells using cell culture inserts from ibidi (Martinsried, GE). Cells were plated into a 12-well plate at a density of 2.0 × 10^5^ cells per well in serum-free medium and incubated overnight at 37°C and 5% CO_2_. The inserts were then removed to create the “wound” between two adherent confluent monolayers of cells. These were washed with PBS before being replenished with experimental medium containing 5% FBS and 0–1 μM of NSL-YHJ-2-27 for 48 h. At 0, 24, 48, and 72 h bright-field images of distinct areas along the “wound” in each of the triplicates were captured using a Nikon Eclipse microscope. A total of nine images were taken for each concentration at each time point. NIS-Elements AR version 4.30 was used to determine the number of cells that had migrated into the “wound” area. The number of migrated cells was then plotted against treatment concentrations using GraphPad Prism software.

### Effect of PCAIs on 3D spheroids invasion

MIA PaCa-2 and PANC-1 cells were suspended in complete medium, seeded at a density of 1.0 × 10^4^ cells per well with 200 μL growth medium in 96U Nunclon Sphera plates (Thermo Scientific, Waltham, MA, USA), and incubated at 37°C/5% CO_2_ for 72 h to form spheroids. Half of the medium was removed, and the remainder was treated with 0 to 10 μM NSL-YHJ-2-27 contained in 1 μL. Matrigel (Corning, NY, 100 μL) mixed with 0 to 10 μM NSL-YHJ-2-27 was added followed by incubation at 37°C/5% CO_2_ for 30 min for the Matrigel to solidify. Images were immediately taken and every 24 h thereafter for 7 days using the Nikon Eclipse microscope. Time-dependent changes in spheroid invasion areas were measured for the control and treated spheroids using NIS- Elements AR version 4.30. The invasion areas for spheroids of PANC-1 and MIA PaCa-2 cells were determined by subtracting the spheroid area of each treatment concentration at the onset from the respective spheroid area at each time point. Graphs showing invasion areas against each time point were plotted using GraphPad Prism version 8 on windows.

### Effect of PCAIs on actin filaments

The impact of PCAIs on cytoskeletal F-actin was examined using Alexa FluorTM568 phalloidin (Cat# A12380). Both MIA PaCa-2 and PANC-1 (1 × 10^4^ cells per well) were plated on an Ibidi 8-well μ slide plate and incubated for 24 h to adhere. The medium was replaced with treatment medium containing 0 μM, 0.1 μM, 0.25 μM, and 0.5 μM of NSL-YHJ-2-27. The treatment media were replaced after 24 h followed by further incubation for 24 h. The cells were fixed with 4% formaldehyde for 5 mins, permeabilized with 0.5% Triton X100 for 15 mins and stained with a mixture of 1X Hoechst stain (DAPI) and 1X Alexa FluorTM568 phalloidin (Cat# A12380). A Keyence BZ-X800 series microscope was used to capture fluorescent images of the F-actin cytoskeleton at a 40X magnification. Fluorescent intensities of the actin filaments and individual cell areas for 100-350 cells for each treatment triplicate were obtained using the Keyence BZ-X800 analyzer.

### Effect of PCAIs on 3D spheroids

To determine the effects of PCAIs on 3D spheroids, MIA PaCa-2 and PANC-1 cells were suspended in complete medium, seeded at a density of 1.0 × 10^4^ cells per well in 96U Nunclon Sphera plates (Thermo Scientific, Waltham, MA, USA), and incubated at 37°C/5% CO_2_ for 72 h for spheroids to form. After 72 h, the spheroids were treated with 0–10 μM NSL-YHJ-2-27 in acetone (0.2% final concentration). Images were taken after 30 mins of treatment and every 24 h thereafter. At 96 h, the spheroids were stained with a mixture of Acridine Orange /Ethidium Bromide (AO/EB, 5 μL of a 100 μg/mL solution) for 30 s and imaged immediately using the Nikon Ti Eclipse microscope. The fluorescence intensities were determined with NIS- Elements AR version 4.30.

### Effects of PCAIs on caspase activity

Apoptosis in cells is primarily induced by active caspases 3 and 7 levels. The CaspaTag™ caspase-3/7 *in situ* fluorescein kit was used to determine the PCAIs effect on caspase activation. PANC-1 (1 × 10^5^ cells per well) were seeded onto an 8-well μ slide plate (ibidi) and allowed to adhere overnight as per the manufacturer’s instructions. After 24 h, the media were replaced with treatment media containing 0, 0.5, 1, 3, and 5 μM of NSLYHJ-2-27. The treatment was repeated after a 24 h followed by further incubation for 24 h. The experimental media were replaced with a 1:30 dilution of the CaspaTag FLICA reagent followed by incubation for 1 h in 5% CO_2_ at 37°C. The CaspaTag FLICA reagent dissolved in media was then removed, and cells were washed using 2 mL of wash buffer. The Keyence BZ-X800 microscope was used to capture images to determine the active caspase levels in cancer cells by quantifying the fluorescence intensities. The BZ-800 analyzer was used to process images and quantify the cellular fluorescence intensities. Graphs showing the effect of PCAIs on caspase activity through mean fluorescence intensities per cell were plotted against concentration using GraphPad Prism 8.4.3.

### Biochemical processes affected by PCAIs

Below is an illustration that provides a summary to the effects of PCAIs treatment on biological characteristics of pancreatic cancer cells.

### Statistical analysis

Unless described otherwise, One-Way ANOVA with Dunnett’s posthoc test was used to determine statistical significance. The values of each treatment group were compared to the respective controls using GraphPad Prism version 8.0 for Windows (San Diego, CA, USA) and ≤0.05 was considered significant.

## CONCLUSIONS

In summary, the limited number of drugs to treat KRAS-driven cancers remains a significant healthcare problem, with the current drugs becoming ineffective due to intrinsic resistance. In view of this, novel therapies are needed to combat the KRAS conundrum. Our study reports on the effectiveness of the PCAIs against two pancreatic cancer cell lines with different *KRAS* mutations, demonstrating their potential pan-mutant KRAS-driven cancer therapeutic applicability. The anticancer mechanism mediated through the MAPK and PI3K/AKT pathways, disruption of the actin cytoskeleton and focal adhesions, cell migration and invasion are in sync with expectations for the PCAIs design to mimic and disrupt the actions of G-proteins that are modified through a polyisoprenyl mono-substitution of a C-terminal cysteine. This demonstrates that targeted disruption of the polyisoprenyl-dependent G-protein-protein interactions is an encompassing therapeutic approach to manage neoplasms with mutation and/or overexpression G-protein-driven cancer progression.
